# A Rare Case of Intracavitary Cardiac Metastasis of Endometrial Carcinosarcoma

**DOI:** 10.7759/cureus.25583

**Published:** 2022-06-01

**Authors:** Masi Javeed, Raghav Ravuri, Zaydi Javeed, Shawn Taylor, Rami Akel

**Affiliations:** 1 Internal Medicine, HCA Florida Bayonet Point Hospital, Hudson, USA; 2 Transitional Year, HCA Florida Bayonet Point Hospital, Hudson, USA; 3 Cardiology, HCA Florida Bayonet Point Hospital, Hudson, USA; 4 Interventional Cardiology, HCA Florida Bayonet Point Hospital, Hudson, USA

**Keywords:** echocardiography in cardio-oncology, port catheter, angio vac, transesophageal echo, uterine neoplasm

## Abstract

A 59-year-old female, with past medical history including endometrial carcinosarcoma with a port-a-cath device, presented due to shortness of breath. Transesophageal echocardiogram demonstrated a mass extending from the right atrium, involving the tricuspid valve, and extending into the right ventricle. Our differential diagnosis included thrombus as well as endocarditis and malignancy; a thrombus was considered to be the most likely etiology due to the port-a-cath device. Use of the novel AngioVac mechanical aspiration device (AngioDynamics, 2021) allowed removal of the mass as well as evaluation by pathology, establishing the unlikely diagnosis of metastatic endometrial carcinosarcoma.

## Introduction

Endometrial carcinosarcomas are rare tumors that account for less than 5% of all endometrial malignancies. They are considered a high-risk variant of endometrial adenocarcinoma [1]. Most carcinosarcomas are visualized as exophytic lesions with no evidence of invasive growth. Causes of this malignancy include nulliparity, advanced age, obesity, exposure to exogenous estrogens, and long-term use of tamoxifen. While hysterectomy with bilateral salpingo-oophorectomy remains the mainstay treatment, there is a need for lymphadenectomy and postoperative adjuvant treatment [2]. This is due to the fact that they have a high rate of recurrence and metastasis. However, cardiac metastasis of endometrial carcinosarcoma is very rare.

## Case presentation

A 59-year-old female, with past medical history including endometrial carcinosarcoma status-post total hysterectomy, bilateral salpingo-oophorectomy, pelvic lymphadenectomy, as well as chemotherapy and immunotherapy, presented to the hospital for chief complaint of shortness of breath. Significant vitals included tachycardia with a heart rate of 107 beats per minute. Physical exam was unremarkable. She had a port-a-cath device on her right upper chest. Notable laboratory values showed hemoglobin of 9.7 grams per deciliter, white blood cell count of 3,800 cells per microliter, and platelet count of 54 per microliter. Serial troponins were unremarkable. 

**Figure 1:**
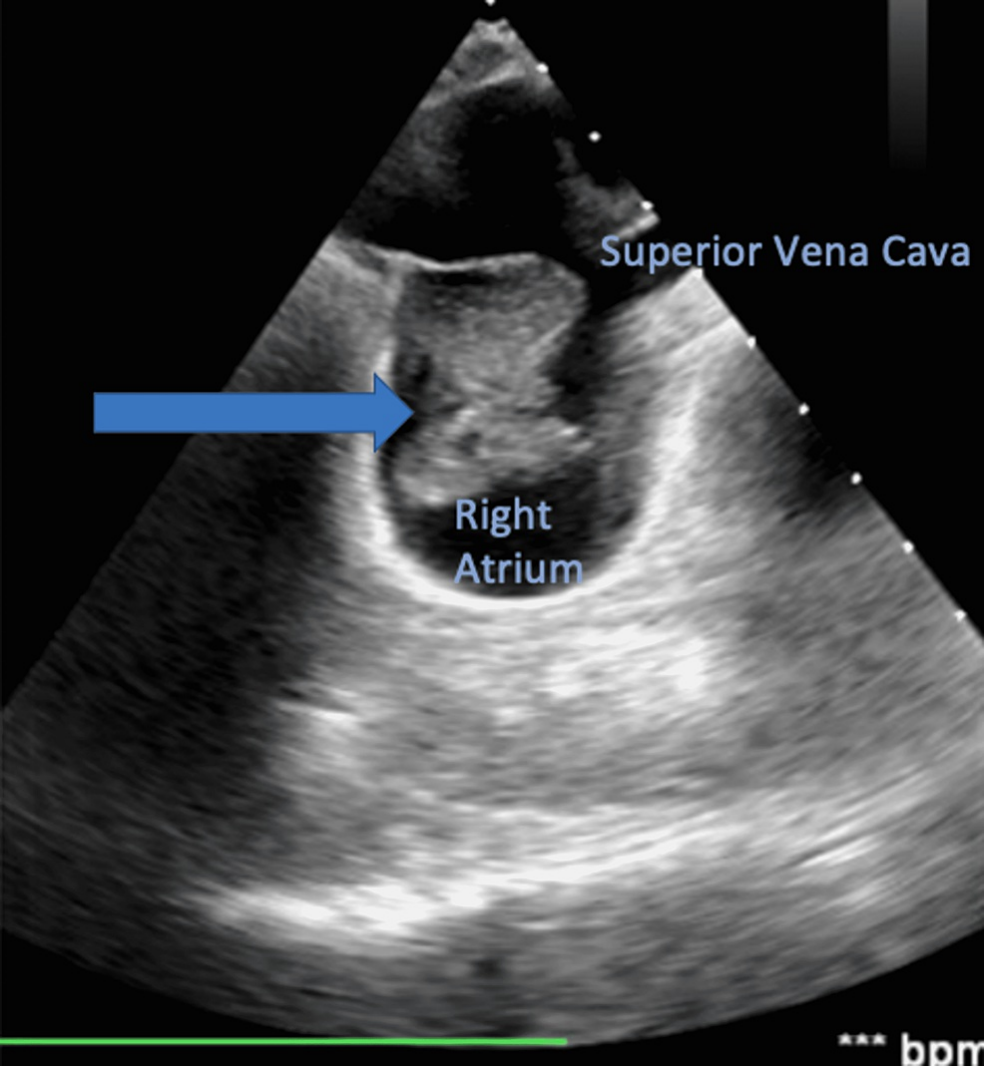
Mid-esophageal bicaval view showed the highly mobile mass to be extending from the right atrium; the mass is marked by the arrow

**Figure 2:**
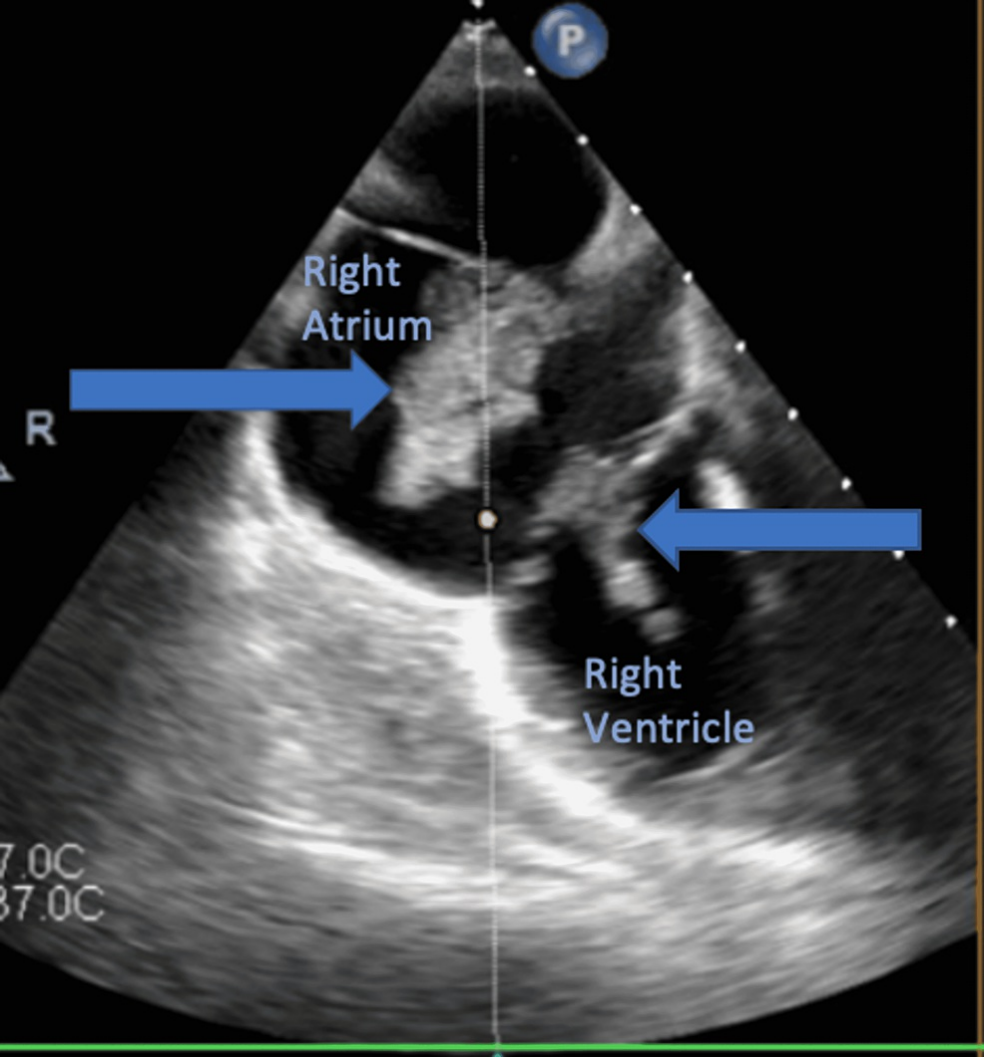
Mid-esophageal view with omniplane angle of zero degrees showed the highly mobile mass extending from the right atrium, involving tricuspid valve, and extending to the right ventricle; the mass is marked by the arrows

Transthoracic echocardiogram showed a large, protruding, echogenic, highly mobile mass involving the right atrium, tricuspid valve, and right ventricleas well as mild right atrial dilation. Transesophageal echocardiogram subsequently showed a large, highly mobile mass extending from the right atrium, involving the tricuspid valve, and extending into the right ventricle as well as moderate tricuspid regurgitation (Figures 1, 2).

We had a discussion with the patient with regard to the mass and the treatment plan. The mass was primarily suspected to be thrombus due to the patient's port-a-cath device. The catheter was removed. The plan was to order anticoagulation and repeat echocardiogram in four weeks. If the mass did not improve or resolve, the plan was to perform aspiration of the mass through the AngioVac system (AngioDynamics, 2021). However, the patient was admitted three weeks later for worsening shortness of breath despite therapeutic anticoagulation and no embolization. Therefore, aspiration of the mass through the AngioVac system was performed. 

Ultrasound-guided access was obtained to the right and left common femoral veins. Therapeutic dose of heparin was given to maintain an activated clotting time more than 300. Serial dilation to a 19-French return cannula was inserted to the left common femoral vein. The right femoral vein was dilated to a 26-French sheath. The AngioVac inflow catheter was advanced to the junction of the inferior vena cava and the right atrial junction. Then, the cannula was de-aired and cardiopulmonary bypass was initiated with gradual increasing in the flow up to 3 liters per minute. Under fluoroscopy and the guidance of transechocardiogram, the AngioVac cannula was advanced to mid-right atrium towards the mass and there was successful removal of the mass. Repeat transesophageal echocardiogram confirmed no damage to the tricuspid valve or worsening of known tricuspid regurgitation. Systems were removed and hemostasis was obtained using figure-of-8 sutures.

The mass removed was pancytokeratin, anti-cytokeratin (CAM) 5.2, desmin, vimentin, S100, smooth muscle antigen, and glypican 3 positive. This led pathology to diagnose the mass as metastatic endometrial carcinosarcoma. 

The patient’s dyspnea improved after the AngioVac procedure. She was discharged with instructions to follow up with primary care, cardiology, and hematology-oncology in the outpatient setting. Unfortunately, the patient’s condition continued to decline over the next few months with metastasis to the spine as well resulting in cord compression and paraplegia. After the patient discussed her situation with her family, she elected for hospice care.

## Discussion

A report by Greenwald et al. showed 6 of 1,100 gynecological cancer cases to have metastasis to the heart, but none was endometrial in origin [3]. Since then, there have been very few reported cases of cardiac metastasis from endometrial cancer: only 24 between 2004 and 2014. Among these cases of metastasized endometrial cancers, the majority are found to be squamous cell carcinoma (50%), leiomyosarcoma (21%), and then adenocarcinoma (17%) followed by others [4]. Endometrial carcinosarcoma is a high-risk variant of adenocarcinoma and, therefore, is even more rare.

Solitary cardiac metastases are very rare and so they usually present along with widespread malignancy. In regards to the heart, the pericardium is the most common site of metastasis followed by the myocardium, epicardium, and interventricular septum. Intracavitary masses are very rare and are usually right-sided. Most common primary tumor origins of cardiac metastases include melanoma, lung, breast, and lymphoma [5]. 

Cardiac metastases have been reported to occur in as little as 1.5% of cancer patients, with even fewer being from gynecologic malignancies and with most cases only discovered on autopsy. This is due to the clinically silent nature of these metastases. The most common symptoms when present include chest pain, arrhythmias, cardiac tamponade, recurrent pulmonary emboli, cardiac failure, and pulmonary hypertension [5]. Echocardiography is the first-line imaging modality in the detection and evaluation of cardiac tumors with the ability to observe location, shape, mobility, and other features. Transesophageal echocardiogram has higher diagnostic accuracy than transthoracic. If possible, cardiac magnetic resonance imaging is even better however due to its ability to observe soft tissue characteristics of the tumor and due to its unobstructed view. Secondary cardiac tumors usually present in the pericardium with irregular surface texture and potentially cause pericardial effusion. Although imaging is helpful, evaluation by pathology remains the definitive method of differentiating neoplastic from non-neoplastic cardiac masses [6].

There is no established treatment for cardiac metastasis of endometrial carcinosarcoma; this is likely due to very low incidence as well as very poor prognosis as the life expectancy of these patients becomes less than six months on average [5]. Combinations of aggressive treatments including open surgical excision, chemotherapy, and radiation therapy have been noted to only increase lifespan by as little as three months and 13 months at most [7]. In our case, the decision to use the AngioVac system to remove the mass was made in order to alleviate symptoms as well as determine the etiology of the mass.

AngioVac is the first aspiration thrombectomy device capable of removing a large burden of undesired intravascular material such as thrombus, foreign body, or even a tumor without the need of thrombolytics. This novel technology has been used successfully in the management of acute and subacute iliocaval thrombosis, catheter-related central venous thrombus, atrial tumors, atrial thrombi, valve vegetations, and submassive and massive pulmonary emboli [8]. The major advantage of using AngioVac is its ability to remove whole intact masses, thus decreasing the amount of post-procedure complications. Several retrospective studies in the literature show that AngioVac had intraprocedural success, with 70% removal of mass on average, in 60% of patients with a right-sided heart mass [8]. One recent prospective study was the Registry of AngioVac Procedures in Detail (RAPID) study. AngioVac was used on patients with caval thromboemboli, right heart masses, pulmonary emboli, and catheter-related thrombi. There was about 70% to 100% mass removal in about 60% of patients with right heart masses. Complications were low and the study concluded that AngioVac is a safe and effective method of cardiac mass removal [9].

However, most studies do not include long-term followup data. In addition, prospective studies comparing the intraoperative success rate of AngioVac to surgical or medical management are still lacking in the literature. Such studies would be helpful in guiding providers to the most appropriate treatment selection on a case-by-case basis in the future. 

## Conclusions

Patient originally presented for shortness of breath, in the setting of known endometrial carcinosarcoma. These symptoms were likely caused by the mass in the right atrium, involving the tricuspid valve, and extending into the right ventricle.

This was a unique case in which the most likely diagnosis of a mass in the presence of port-a-cath device was thrombus. However, by the use of the AngioVac system, we were able to successfully remove the mass and, through histopathology, metastatic endometrial carcinosarcoma was confirmed.
